# Estimated glomerular filtration rate and daily amount of urinary protein predict the clinical remission rate of tonsillectomy plus steroid pulse therapy for IgA nephropathy

**DOI:** 10.1007/s10157-013-0867-8

**Published:** 2013-09-20

**Authors:** Keisuke Suzuki, Naoto Miura, Hirokazu Imai

**Affiliations:** Division of Nephrology and Rheumatology, Department of Internal Medicine, Aichi Medical University School of Medicine, Nagakute, Aichi 480-1195 Japan

**Keywords:** Clinical remission, Heat map, IgA nephropathy, Tonsillectomy plus steroid pulse therapy

## Abstract

**Background:**

This retrospective study was designed to estimate the clinical remission (CR) rate of tonsillectomy plus steroid pulse (TSP) therapy in patients with IgA nephropathy.

**Methods:**

Based on 292 of 302 patients with IgA nephropathy treated at 11 Japanese hospitals, we constructed heat maps of the CR rate at 1 year after TSP with the estimated glomerular filtration rate (eGFR), grade of hematuria, pathological grade, number of years from diagnosis until TSP, and age at diagnosis on the vertical axis and the daily amount of urinary protein (urinary protein) on the horizontal axis. We compared subgroups usinge Student’s *t* test, the chi-square test with Yates correction, or Fisher’s exact probability test.

**Results:**

The first heat map of eGFR and urinary protein showed that the CR rate was 71 % (CR vs. non-CR, 96 vs. 40) in patients with eGFR greater than 30 ml/min/1.73 m^2^ and 0.3–1.09 g/day of urinary protein. However, the CR rate in patients with more than 1.50 g/day of urinary protein was approximately 30 %. The second heat map of grade of hematuria and urinary protein revealed that the CR rate is 72 % (CR vs. non-CR, 93 vs. 37) in patients with more than 1+ hematuria and 0.3–1.09 g/day of urinary protein; however, it was 28.6 % in patients with no hematuria. The third heat map of pathological grade and urinary protein demonstrated that the highest CR rate was 83 % (CR vs. non-CR, 52 vs. 11) in patients with pathological grade I or II disease and less than 1.09 g/day of urinary protein, as opposed to 22 % (CR vs. non-CR, 9 vs. 32) in patients with pathological grade III or IV disease and more than 2.0 g/day of urinary protein. The fourth heat map of the number of years from diagnosis until TSP and urinary protein revealed that the former did not influence the CR rate in patients with less than 1.09 g/day of urinary protein. However, in patients with more than 1.10 g/day of urinary protein, the CR rate of the subgroup with less than 6 years was 43 % (CR vs. non-CR; 23 vs. 54) compared to 23 % (CR vs. non-CR, 11 vs. 48; *P* = 0.01) in the subgroup with more than 6 years. The fifth heat map of age at diagnosis and urinary protein showed that the CR rate is approximately 72 % (CR vs. non-CR, 73 vs. 28) in patients older than 19 years at diagnosis with 0.3–1.09 g/day of urinary protein.

**Conclusions:**

The daily amount of urinary protein is an important predictor of the CR rate after TSP in IgA nephropathy patients. Heat maps are useful tools for predicting the CR rate associated with TSP.

## Introduction

In 2001, Hotta et al. [1] proposed tonsillectomy plus steroid pulse (TSP) as a new approach that can induce clinical remission (CR) in IgA nephropathy patients. The profile of 329 patients in their retrospective study was as follows: age (mean ± SD), 36.1 ± 12.8 years; daily proteinuria, 1.40 ± 1.09 g; serum creatinine, 1.14 ± 0.48 mg/dl. In a Cox regression analysis with 13 variables, serum creatinine <1.3 mg/dl, daily proteinuria between 0.5 and 1.5 g, histological score (index of glomerular lesion, calculated by the degree of mesangial proliferation and sclerosis) <2.00, steroid pulse therapy, and tonsillectomy were identified as prognostic factors for CR. Recently, a subsequent analysis revealed that each year 600 patients in Japan received TSP in 2006 [2]. In 2010, more than 1,000 patients per year received TSP in Japan, with half achieving CR, defined as no urinary abnormalities, 1 year after treatment. In a retrospective multicenter study, Miura et al. found that 54.1 % of patients reached CR at 1 year after TSP. There were significant differences between patients who reached CR and those who did not reach CR in terms of the number of years from diagnosis until TSP (*P* = 0.02), daily proteinuria (*P* < 0.0001), serum creatinine (*P* = 0.006), and pathological grade (*P* = 0.0006). Multivariate logistic regression analysis demonstrated that factors associated with resistance to TSP include young age, massive amounts of urinary protein, absence of hematuria, and severe pathological grade. Our present study was designed to clarify the indications and limitations of TSP for IgA nephropathy patients and to clarify whether a heat map, by using several factors on vertical axis and daily amount of urinary protein on horizontal axis, can predict CR.

## Methods

The present retrospective multicenter study was approved by the Ethics Committee of Aichi Medical University and was designed as a sub-analysis of previously reported data.

### Patients

From our previous study involving 303 patients [2], 292 with sufficient laboratory data such as the daily amount of urinary protein and serum creatinine values were analyzed here. The present study included 128 males and 164 females, whose mean age was 34.17 ± 13.75 years (range, 12–73). The mean duration from diagnosis to TSP was 6.1 ± 6.1 years. The daily amount of urinary protein was 1.10 ± 1.29 g, and the serum creatinine level was 0.93 ± 0.38 mg/dl. There were 14, 47, 74, and 157 patients with hematuria grade 0, 1+, 2+, and 3+, respectively. The distribution of pathological grade was: I, 14 patients; II, 57 patients; III, 120 patients; IV, 101 patients. The prevalence of antihypertensive medication use was 41.6 %. The CR rate at 1 year after TSP was 55.5 %. Previous studies using multivariate logistic regression have identified several factors that predict resistance to TSP such as age at diagnosis, daily amount of urinary protein, hematuria, and pathological grade. The use of angiotensin-converting enzyme inhibitors or angiotensin II receptor blockers and gender had no impact on CR in previous studies.

### The definition of CR

CR was determined based on urinary analysis, as described in a previous report [2]. Remission of proteinuria was defined as negative (−) or trace (±) proteinuria on the urine dipstick test, while remission of hematuria was specified as the absence of blood on the dipstick test and urinalysis. CR was defined as the complete resolution of both proteinuria and hematuria.

### Estimation of the glomerular filtration rate (GFR)

The estimated GFR (eGFR) was calculated using the Japanese equation [3]:

$${\text{eGFR (ml/min/1}}{\text{.73}}\,{{\text{m}}^2}) = 194 \times {\text{C}}{{\text{r}}^{ - 1.094}} \times {\text{ag}}{{\text{e}}^{ - 0.287}} \times (0.739\,{\text{if}}\,{\text{female}})$$

### Pathological grade

Pathological grade of the kidney biopsy was assessed by pathologists or nephrologists at each participating hospital during using the previous criteria from the Joint Committee of the Research Group on Progressive Renal Diseases and the Japanese Society of Nephrology [4].

Biopsy samples were graded based on the following criteria:

Grade I: Glomerular findings: Slight mesangial cell proliferation and increased matrix. Glomerulosclerosis, crescent formation, or adhesion to Bowman’s capsule is not observed. Interstitial and vascular findings: Prominent changes are not seen in the interstitium, renal tubuli, or blood vessels.

Grade II: Glomerular findings: Slight mesangial cell proliferation and increased matrix. Glomerulosclerosis, crescent formation, or adhesion to Bowman’s capsule seen in <10 % of all biopsied glomeruli. Interstitial and vascular findings: Prominent changes are not seen in the interstitium, renal tubuli, or blood vessels.

Grade III: Glomerular findings: Moderate, diffuse mesangial cell proliferation and increased matrix. Glomerulosclerosis crescent formation or adhesion to Bowman’s capsule seen in 10–30 % of all biopsied glomeruli. Interstitial and vascular findings: Cellular infiltration is slight in the interstitium except around some sclerosed glomeruli. Tubular atrophy is slight, and mild vascular sclerosis is observed.

Grade IV: Glomerular findings: Severe, diffuse cell proliferation and increased matrix. Glomerulosclerosis, crescent formation, or adhesion to Bowman’s capsule seen in >30 % of all biopsied glomeruli. When sites of sclerosis are totaled and converted to global sclerosis, the sclerosis rate is >50 % of all glomeruli. Some glomeruli also show compensatory hypertrophy. The sclerosis rate is the most important of these indices. Interstitial and vascular findings: Interstitial cellular infiltration and tubular atrophy, as well as fibrosis are seen. Hyperplasia or degeneration may be seen in some intrarenal arteriolar walls.

### Construction of the CR rate heat maps

Clinical remission was shown as “C” and non-clinical remission as “N.” The CR rate was calculated in each cell. Cells were color coded by the CR rate with >66 % represented by dark blue, 50–65 % by light blue, 50 % by yellow, 33–49 % by orange, <33 % by dark red, and patient number zero by white.

**Fig. 1:**
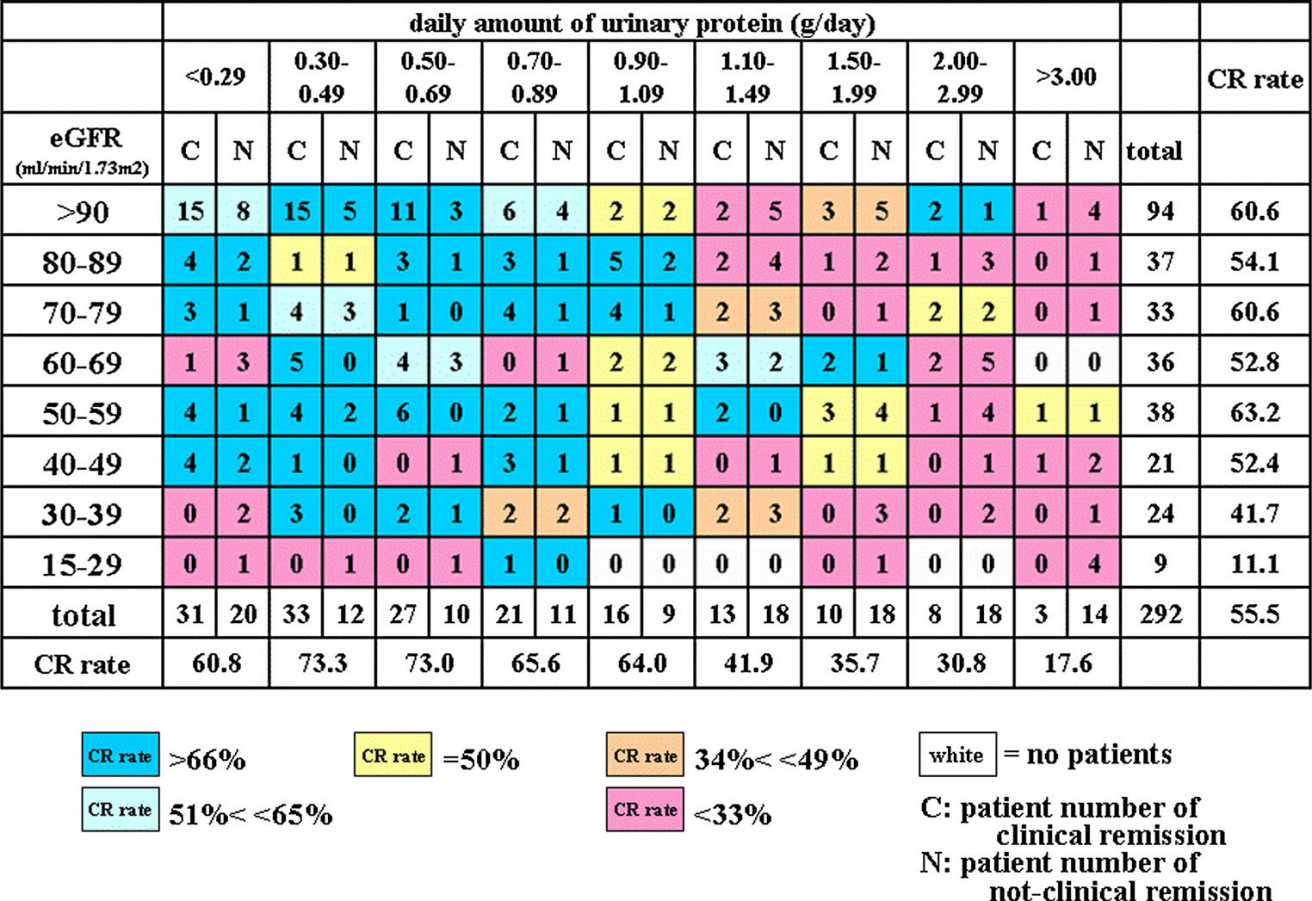
A heat map of the CR rate based on the eGFR value and daily amount of urinary protein. A gradation from *dark blue* in the *upper left* corner to *dark red* in the *lower right* corner is observed. A relatively high CR rate of 71 % (CR vs. non-CR, 96 vs. 40) was observed in patients with eGFR greater than 30 ml/min/1.73 m^2^ and 0.3–1.09 g/day of urinary protein. On the other hand, the CR rate in patients with more than 1.50 g/day of urinary protein was 29.6 % (CR vs. non-CR, 21 vs. 50). The CR rate in patients with hematuria alone (<0.29 g/day of urinary protein) was relatively low at 60.8 % (CR vs. non-CR, 31 vs. 20), compared to 73 % (CR vs. non-CR, 60 vs. 22) in patients with 0.3–0.69 g/day of urinary protein (*P* = 0.19). Patients with <0.29 g/day of urinary protein and eGFR of 60–69 ml/min/1.73 m^2^ have a low CR rate; however, there is no significant difference among these subgroups

**Fig. 2:**
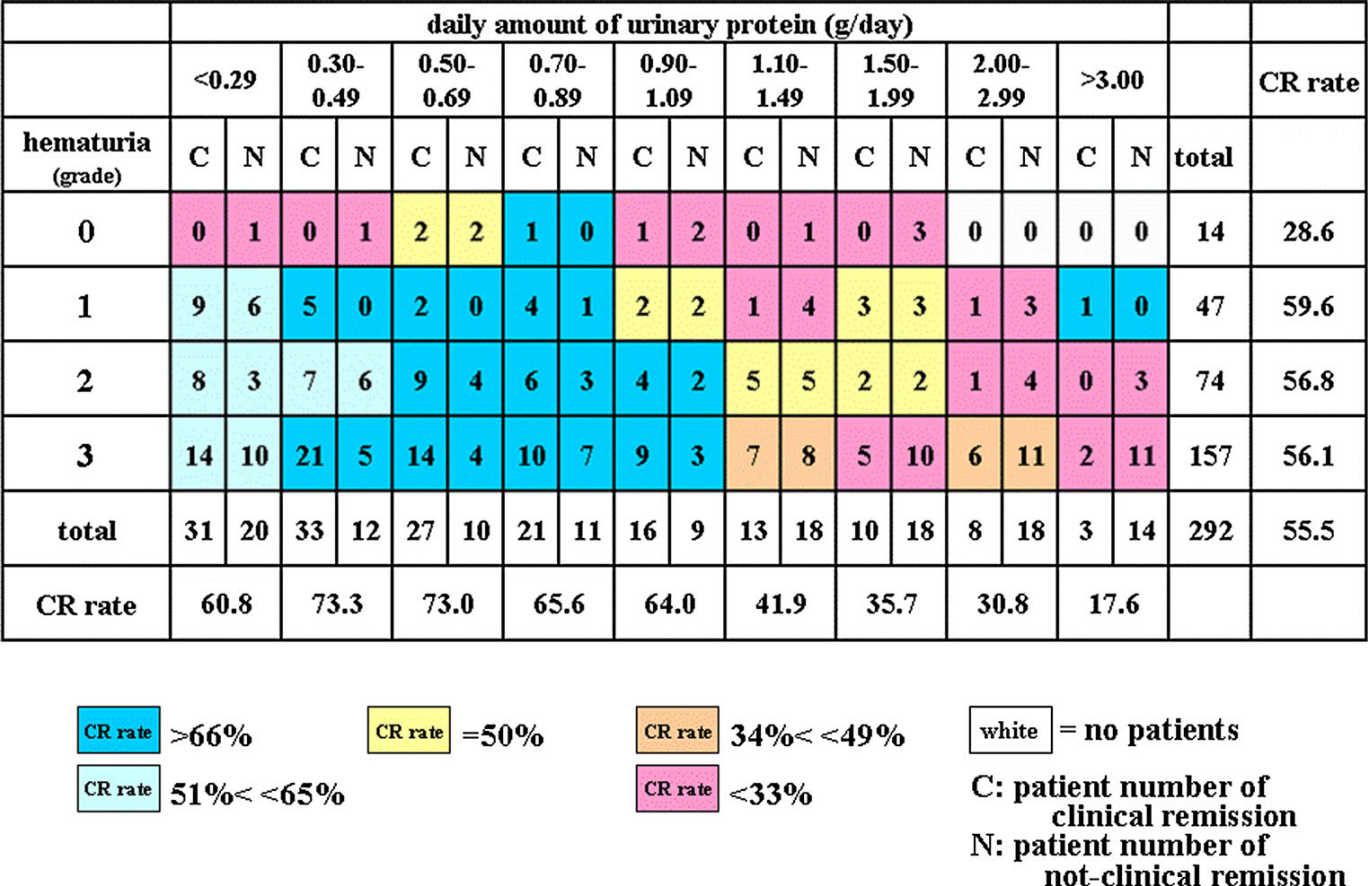
A heat map of the CR rate based on the grade of hematuria and daily amount of urinary protein. A graduation from *dark blue* in the *upper left* corner to *dark red* in the *lower right* corner is observed. Patients with no hematuria had a worse CR rate, 28.6 % (CR vs. non-CR, 4 vs. 10), compared to subgroups with hematuria (56 %; CR vs. non-CR, 158 vs. 124; *P* = 0.04). The CR rate was 72 % (CR vs. non-CR, 108 vs. 49) in patients with more than 1+ hematuria and 0.3–0.89 g/day of urinary protein. The CR rate was 25.6 % (CR vs. non-CR, 11 vs. 32) in patients with more than 1+ hematuria and more than 2.0 g/day of urinary protein

**Fig. 3:**
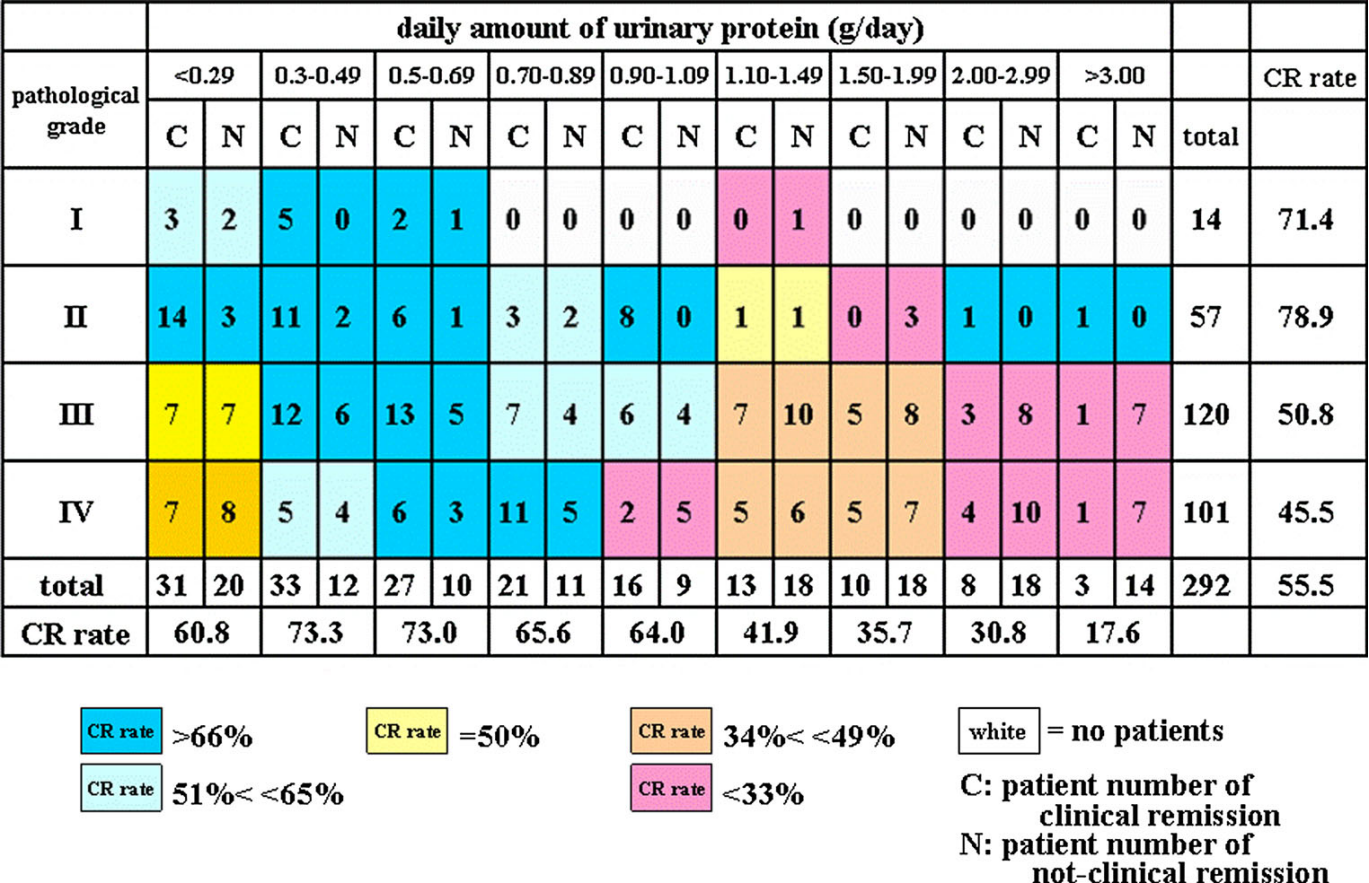
A heat map of the CR rate based on pathological grade and daily amount of urinary protein. A gradation from *dark blue* in the *upper left* corner to *dark red* in the *lower right* corner is observed. The CR rate of patients with pathological grade I or II disease and <1.09 g of daily urinary protein was 82.5 % (CR vs. non-CR, 52 vs. 11). In contrast, the CR rate of patients with pathological grade III or IV disease and more than 2.0 g of daily urinary protein was 28.1 % (CR vs. non-CR, 9 vs. 32; *P* < 0.00001)

**Fig. 4:**
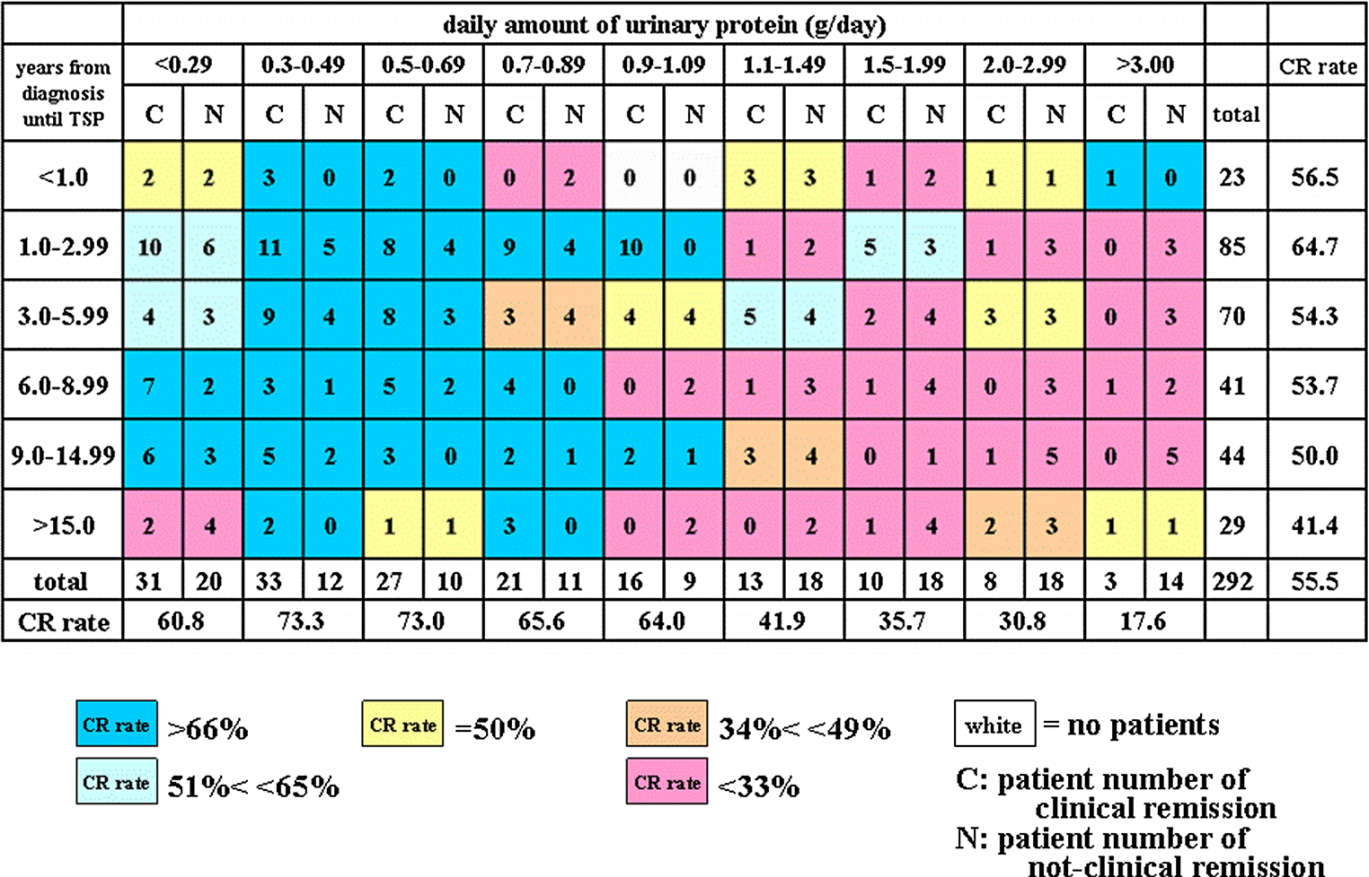
A heat map of the CR rate based on the number of years from diagnosis until TSP and daily amount of urinary protein. A gradation from *dark blue* starting to the *left* of 1.09 g of daily urinary protein to *dark red* on the *right* is observed. In patients with daily urinary protein between 0.3 and 1.09 g, the number of years from diagnosis until TSP did not influence the CR rate, which was in the 70 % range. However, in patients with more than 1.10 g/day of urinary protein, the CR rate of the subgroup with less than 6 years was 43 % (CR vs. non-CR, 23 vs. 54) compared to 23 % in the subgroup with more than 6 years (CR vs. non-CR, 11 vs. 48; *P* = 0.01)

**Fig. 5:**
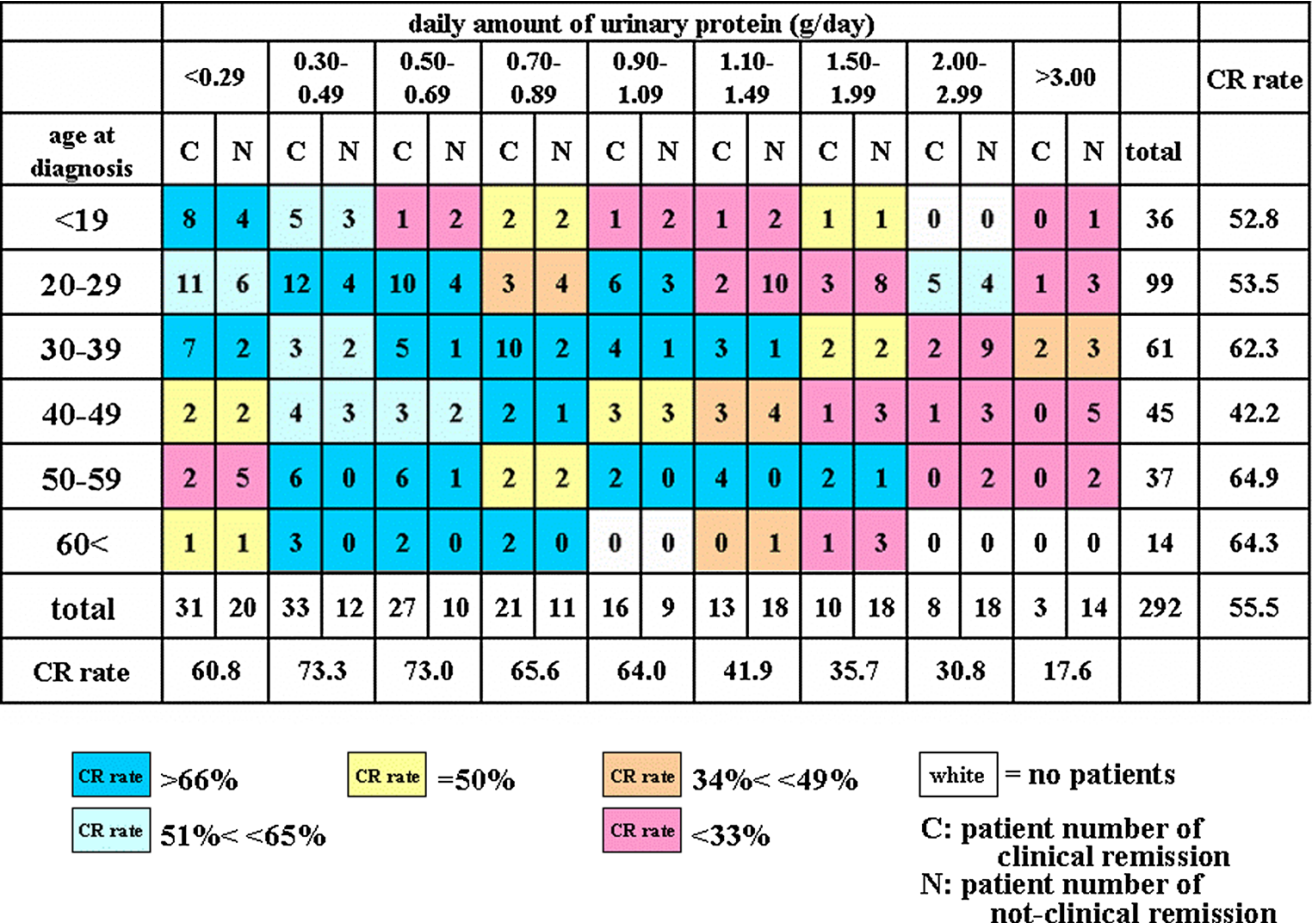
A heat map of the CR rate based on the age at diagnosis and daily amount of urinary protein. A graduation from *dark blue* starting from the *lower left* of 0.3–0.89 g of urinary protein to *dark red* on the *right* is observed. The CR rate was 73 % (CR vs. non-CR, 88 vs. 35) in patients with 0.3–1.09 g/day of urinary protein who were older than 20 years at diagnosis. However, relatively low CR rates of 52.8 and 42.2 % were found in patients <19 years old and between 40 and 49 years old at diagnosis, respectively

The first heat map (Fig. 1) shows the CR rate according to eGFR and urinary protein levels. eGFR, depicted on the vertical axis, was divided into eight subgroups with eGFR >90, 80–89, 70–79, 60–69, 50–59, 40–49, 30–39, and 15–29 ml/min/1.73 m^2^, respectively. Urinary protein was divided into nine subgroups: <0.29, 0.30–0.49, 0.50–0.69, 0.70–0.89, 0.90–1.09, 1.10–1.49, 1.50–1.99, 2.00–2.99, and >3.00 g/day. The second heat map (Fig. 2) has the grade of hematuria on the vertical axis and urinary protein on the horizontal axis. The third heat map (Fig. 3) has the pathological grade on the vertical axis and urinary protein on the horizontal axis. A fourth heat map, with the number of years from diagnosis until TSP on the vertical axis and urinary protein on the horizontal axis, was also constructed (Fig. 4). The number of years from diagnosis until TSP was divided into five subgroups: <1.0, 1.0–2.99, 3.0–5.99, 6.0–8.99, 9.0–14.99, and >15.0 years, respectively. A fifth heat map was constructed using age at diagnosis on the vertical axis and urinary protein on the horizontal axis (Fig. 5). Age at diagnosis was divided into six subgroups: <19, 20–29, 30–39, 40–49, 50–59, and >60 years.

### Statistical analysis

Quantitative values were expressed as mean ± SD, unless otherwise noted. Data comparisons were carried out using Student’s *t* test or the chi-square test with the Yates correction for continuity or Fisher’s exact probability test. *P* values <0.05 were considered statistically significant.

## Results

### The CR rate according to eGFR and urinary protein levels

Figure 1 shows a heat map of the CR rate at 1 year after TSP for IgA nephropathy patients, which demonstrates a gradient from high to low CR rates. There is a significant difference between subgroups with less than 1.09 g/day of proteinuria (CR vs. non-CR, 128 vs. 62) and more than 1.10 g/day (CR vs. non-CR, 34 vs. 68; *P* < 0.00001). A high CR rate of 71 % (CR vs. non-CR, 96 vs. 40) was observed in patients with eGFR levels greater than 30 ml/min/1.73 m^2^ and 0.3–1.09 g/day of urinary protein. On the other hand, the CR rate in the subgroup with more than 1.50 g/day of urinary protein was 29.6 %. In contrast, the CR rate was as low as 60.8 % in patients with hematuria alone (<0.29 g/day of urinary protein; CR vs. non-CR, 31 vs. 20) compared to 73 % in patients with 0.3–0.69 g/day of urinary protein (CR vs. non-CR, 60 vs. 22; *P* = 0.19). Patients with <0.29 g/day of urinary protein and 60–69 ml/min/1.73 m^2^ of eGFR had a low CR rate, but there was no significant difference.

### The CR rate according to the grade of hematuria and urinary protein

Figure 2 shows that the CR rate was 72 % (CR vs. non-CR, 108 vs. 49) in patients with more than 1+ hematuria and 0.3–0.89 g/day of urinary protein; however, the CR rate was 28.6 % in patients without hematuria (14 out of 292 patients). The CR rate of the 1+, 2+, and 3+ hematuria subgroups was 59.6, 56.8, and 56.1 %, respectively.

### The CR rate according to pathological grade and urinary protein

Figure 3 demonstrates that the CR rate in patients with pathological grade I or II disease and <1.09 g/day of urinary protein was 82.5 % (CR vs. non-CR, 52 vs. 11), whereas the subgroup with pathological grade III or IV disease and more than 2.0 g/day of urinary protein had a CR rate of 28.1 % (CR vs. non-CR, 9 vs. 32; *P* < 0.00001). The former subgroup had the highest CR rate, while the latter had the lowest CR rate.

### The CR rate according to the number of years from diagnosis until TSP and urinary protein

Figure 4 shows that the number of years from diagnosis until TSP did not influence the CR rate in patients with between 0.3 and 1.09 g/day of urinary protein. The CR rate was approximately 70 % in these subgroups. However, in patients with more than 1.10 g/day of urinary protein, the CR rate of the subgroup with less than 6 years was 43 % (CR vs. non-CR, 23 vs. 54), compared to 23 % for the subgroup with more than 6 years (CR vs. non-CR, 11 vs. 48; *P* = 0.01).

### The CR rate according to the age at diagnosis and urinary protein level

Figure 5 shows that the CR rate was 73 % (CR vs. non-CR; 88 vs. 35) in patients with between 0.3 and 1.09 g/day of urinary protein who were more than 20 years old at diagnosis. However, relatively low CR rates of 52.8 and 42.2 % were found in patients <19 years old and between 40 and 49 years old, respectively.

There was no relationship between the number of years from diagnosis until TSP and pathological grade or eGFR, respectively (data not shown).

## Discussion

This study revealed three major points. The first is that heat maps, based on eGFR and urinary protein, or pathological grade and urinary protein, can predict the CR rate at 1 year after TSP therapy in patients with IgA nephropathy. The second is that urinary protein is an important factor influencing the CR rate among the variables studied, which also included grade of hematuria, pathological grade, number of years from diagnosis until TSP, and age at diagnosis. The third is that patients with proteinuria alone (without hematuria) or hematuria alone (<0.29 g/day of urinary protein) have relatively low CR rates of 28.5 and 60.8 %, respectively.

Heat maps are useful tools for physicians to predict the CR rate in individual patients and to explain the predicted CR rate to patients and their families. The highest CR rate was 82.5 % in patients with pathological grade I or II disease and <1.09 g/day of urinary protein, and approximately 70 % in patients with eGFR >30 ml/min/1.73 m^2^ and <1.09 g/day of urinary protein. These subgroups are good candidates for TSP. On the other hand, a poor CR rate of approximately 30 % was observed in patients with more than 1.5 g/day of urinary protein regardless of eGFR. A randomized controlled trial comparing TSP, steroid pulse therapy, and antiplatelet drugs is needed to clarify the best treatment for IgA nephropathy patients with <1.09 g/day of urinary protein, because observations on long-term outcomes of IgA nephropathy with minimal or no proteinuria have revealed that 37.5 % of patients reach CR after a median of 48 months [5].

Recently, Ieiri et al. [6] emphasized that a shorter duration from diagnosis until TSP is associated with a high likelihood of CR in IgA nephropathy patients treated with TSP. In our previous study, the comparison between patients who reached CR and those who did not reach CR revealed significant differences in the number of years from diagnosis until TSP (*P* = 0.02), daily proteinuria (*P* < 0.0001), serum creatinine (*P* = 0.006), and pathological grade (*P* = 0.0006). However, multivariate logistic regression analysis did not identify the number of years from diagnosis until TSP as a predictive factor. The present study also revealed that the number of years from diagnosis until TSP does not necessarily influence the CR rate; when patients have between 0.3 and 1.09 g/day of urinary protein, the CR rate is approximately 70 %, independent of the number of years from diagnosis until TSP. On the other hand, the number of years form diagnosis until TSP is an important factor in patients with more than 1.1 g/day of urinary protein, because the CR rate was 23 % in patients with more than 6 years from diagnosis until TSP compared to 43 % in patients with <6 years from diagnosis until TSP (*P* = 0.01). The above results suggest that urinary protein is a more essential predictive factor than the number of years from diagnosis until TSP.

Regarding resistance to TSP, based on multivariate logistic regression analysis we previously reported that resistance to TSP therapy depends on age at diagnosis, urinary proteinuria, grade of hematuria, and pathological grade [2]; namely, young age and the absence of hematuria are associated with resistance to TSP. Recently, Ieiri et al. [6] also pointed out that higher age has a favorable impact on the CR rate after TSP. With regards to hematuria, the present study demonstrated that the CR rate in patients with no hematuria (14 out of 292 IgA nephropathy patients) is only 28.6 % compared to 59.6, 56.8, and 56.1 % in patients with 1+, 2+, and 3+ hematuria, respectively. Extensive review of the literature on the relationship between TSP and hematuria revealed no studies except for our previous report [2]. IgA nephropathy patients without hematuria may have nephrosclerosis or hereditary nephritis with concomitant glomerular IgA deposition, because 4 % of normal persons without urinary abnormalities are reported to have glomerular IgA deposition on postmortem examination after accidental death [7]. Concomitant glomerular IgA deposition has been reported in hereditary nephritis, including thin basement membrane disease [8–10], mild Alport syndrome [11], focal segmental glomerulosclerosis [12], and complement factor abnormalities [13]. Moreover, the CR rate in patients without proteinuria (mainly hematuria alone) is relatively low, 60.8 % compared to approximately 73.0 % in patients with 0.3–0.69 g/day of urinary protein. TSP hardly induces CR in these patients of combination with hereditary nephritis and glomerular IgA deposition. We have to pay attention to the diagnostic criteria of IgA nephropathy when patients show no hematuria or no proteinuria because thin basement membrane disease occurs in up to 9 % of the general population according to an analysis of donor kidney grafts [14], and concomitant glomerular IgA deposition is observed in 4 % of normal population [7].

In conclusion, heat maps with the eGFR or pathological grade and daily amount of urinary protein are useful tools for predicting the CR rate of TSP for IgA nephropathy.
